# Ionization engineering unlocks functional mesoporous polymers for solar-driven uranium extraction

**DOI:** 10.1093/nsr/nwag623

**Published:** 2026-09-29

**Authors:** Xin Xu, Markus Antonietti

**Affiliations:** Department of Colloid Chemistry, Max Planck Institute of Colloids and Interfaces, Germany; Department of Colloid Chemistry, Max Planck Institute of Colloids and Interfaces, Germany

The oceans contain vast uranium that could support the long-term development of nuclear energy. However, extracting uranium from seawater remains challenging because of its extremely low concentration (∼3.3 ppb), competing ions and complex marine environments [1]. Photocatalytic extraction offers an alternative to conventional adsorption by coupling uranium binding with light-driven chemical conversion [2]. Nevertheless, simultaneously optimizing accessible binding sites, molecular functionality and mass transport remains difficult. Although mesoporous architectures facilitate molecular diffusion and accommodate reaction products, incorporating structurally complex functional ligands into ordered polymer frameworks is often hindered by poor precursor solubility, incompatible polymerization kinetics and disrupted self-assembly [3].

In a recent study published in *National Science Review*, the Wei Li group and collaborators report an ionization-regulated self-assembly strategy for constructing mesoporous salen polymer nanospheres for photocatalytic uranium extraction [4]. The key innovative step lies in the control of precursor ionization to balance aqueous solubility, polymerization reactivity and interactions with surfactant micelles. Partial ionization improves the solubility and formaldehyde reactivity of the otherwise poorly soluble salen ligands while preserving sufficient hydrogen-bonding interactions for cooperative assembly. Insufficient ionization prevents effective co-assembly, whereas excessive ionization weakens precursor−template interactions and disrupts mesostructural ordering. This intermediate ionization window presents a breakthrough for the formation of ordered mesoporous frameworks (Fig. 1A).

**Figure 1.**
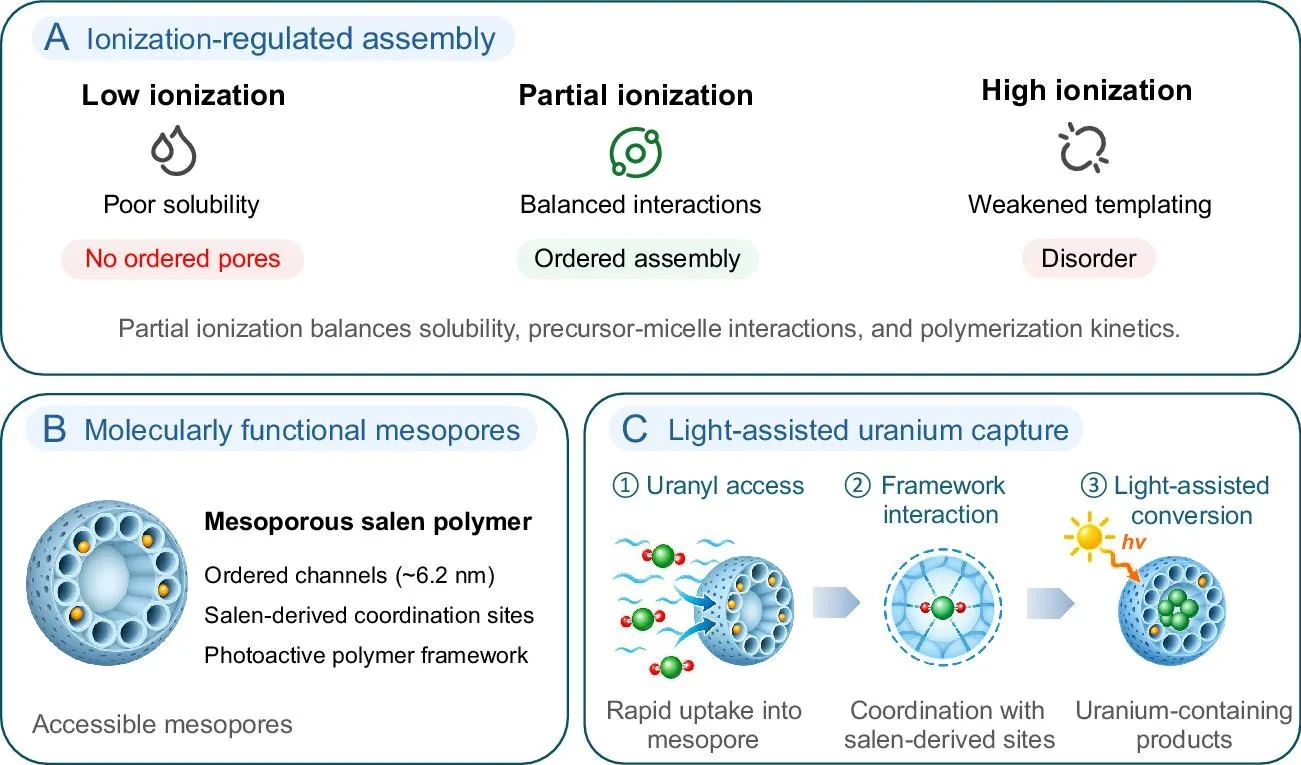
Ionization-regulated assembly of functional mesoporous salen polymers for photocatalytic uranium extraction. (A) Partial ionization balances precursor solubility, polymerization reactivity and interactions with surfactant micelles, enabling ordered mesostructure formation. (B) The resulting mesoporous polymer integrates accessible pore channels, salen-derived coordination motifs and photocatalytic functionality. (C) Schematic illustration of uranyl transport into mesopores, interaction with the salen-derived framework and light-assisted uranium transformation, leading to the formation of uranium-containing products. Adapted conceptually from Chu *et al.* [4].

The resulting polymer nanospheres, denoted mDSR, excitingly possess ordered mesopores of ∼6.2 nm and integrate salen-derived coordination motifs within a photoactive organic framework (Fig. 1B). Notably, the strategy can accommodate several

structurally distinct salen and salen-type ligands, demonstrating a generally modular route to tailoring framework chemistry. By combining accessible mesopores, molecular recognition and photocatalytic functionality, these materials are now able to couple uranium capture with photochemical conversion.

Under simulated sunlight without sacrificial agents, mDSR achieves a high and ultrafast uranium uptake of 867 mg g^−1^ from a 10 mg L^−1^ U(VI) solution within 10 h, compared with only 32 mg g^−1^ under dark conditions [4]. Photophysical measurements and theoretical calculations suggest enhanced photoinduced charge separation and favourable uranyl adsorption energetics. Structural analyses identify uranyl peroxide hydrate [(UO_2_)O_2_·2H_2_O] among the recovered products, while X-ray photoelectron spectroscopy reveals mixed U(VI)/U(IV) oxidation states. These findings support light-assisted uranium transformation (Fig. 1C), although the detailed reaction pathway and fate of transient uranium species remain to be elucidated.

The broader significance of this work, however, lies in extending established self-assembly chemistry to structurally complex functional ligands. Looking ahead, translating these advances into practical seawater uranium recovery will require validation at natural uranium concentrations under prolonged marine exposure. Quantifying binding-site utilization, developing efficient uranium recovery and material regeneration strategies, and establishing long-term resistance to biofouling are the next steps to address. Further engineering of pore connectivity, ligand environments and framework stability, however, makes us expect improvement of these properties. Extending ionization-regulated assembly to diverse molecular building blocks indeed promises new opportunities and insights for chemically programmable mesoporous polymers in selective separation and photocatalysis.
